# Patients' perception and actual practice of informed consent, privacy and confidentiality in general medical outpatient departments of two tertiary care hospitals of Lahore

**DOI:** 10.1186/1472-6939-9-14

**Published:** 2008-09-25

**Authors:** Ayesha Humayun, Noor Fatima, Shahid Naqqash, Salwa Hussain, Almas Rasheed, Huma Imtiaz, Sardar Zakariya Imam

**Affiliations:** 1 Department of Community Health Sciences, FMH College of Medicine and Dentistry, Lahore, Pakistan

## Abstract

**Background:**

The principles of informed consent, confidentiality and privacy are often neglected during patient care in developing countries. We assessed the degree to which doctors in Lahore adhere to these principles during outpatient consultations.

**Material & Method:**

The study was conducted at medical out-patient departments (OPDs) of two tertiary care hospitals (one public and one private hospital) of Lahore, selected using multi-stage sampling. 93 patients were selected from each hospital. Doctors' adherence to the principles of informed consent, privacy and confidentiality was observed through client flow analysis performed by trained personnel. Overall patient perception was also assessed regarding these practices and was compared with the assessment made by our data collectors.

**Results:**

Some degree of informed consent was obtained from only 9.7% patients in the public hospital and 47.8% in the private hospital. 81.4% of patients in the public hospital and 88.4% in the private hospital were accorded at least some degree of privacy. Complete informational confidentiality was maintained only in 10.8% and 35.5% of cases in public & private hospitals respectively. Informed consent and confidentiality were better practiced in the private compared to the public hospital (two-sample t-test > 2, p value < 0.05). There was marked disparity between the patients' perspective of these ethical practices and the assessment of our trained data collectors.

**Conclusion:**

Observance of medical ethics is inadequate in hospitals of Lahore. Doctors should be imparted formal training in medical ethics and national legislation on medical ethics is needed. Patients should be made aware of their rights to medical ethics.

## Introduction

Medical ethics investigate ethical issues arising in medicine and healthcare provision by applying the principles of moral philosophy. Medical ethics are often defined as 'the disciplined study of morality in medicine'[1]. This morality in medicine concerns not only research activities but also the day-to-day medical practice of the doctors vis-à-vis their patients. Ancient ethical codes were often compiled in the form of oaths, the most famous being the Oath of Hippocretes [2]. While the earlier concepts of 'no-harm' and 'best-interest' have been retained, their application has evolved from paternalism into practices of informed consent, privacy and confidentiality that now find their place among the fundamental concepts of medical ethics.

Reports on issues of patient consent can be traced in the US to the early 18^th ^century [3]. These issues were centered on simple rights of the patients in giving approval of their treatment. Further development of this concept has produced the term 'informed consent' which recognizes not only the patients' autonomy in decision but also the right to complete information. The informed consent process requires the physician to explain in sufficient detail, the diagnostic, therapeutic and prognostic reasoning that leads to his expert decision on what is in the best interest of the patient [1,4,5]. Paternalism and coercion are antithetical to the concept of informed consent [1,5].

In one form or the other, respect for privacy and confidentiality has also been a responsibility of the physicians throughout history [6,7]. Recently though, significant attention has been focused on these principles with their formal introduction in most modern codes of ethics and the federal Health Insurance Portability and Accountability Act (HIPAA) in USA. The concepts of privacy and confidentiality are closely related. Privacy is a broader term including physical privacy, informational privacy, protection of personal identity and the ability to make choices without interference [7]. Confidentiality is a narrower term referring to informational privacy and the duty not to disclose any patient information without prior approval from the patient. Privacy and confidentiality are not only basic rights of the patients but also serve to further a trustful, frank and open relationship with the doctor, thus improving patient care [8]. It has also been noted that patients often over- or underestimate their ethical rights in medicine [8,9].

While most western countries have enshrined these concepts of informed consent, privacy and confidentiality in federal or state laws and codes of ethics, such law-making is almost non-existent in Pakistan although there have been some recent efforts to create ethical guidelines for research and medical practice. Significantly, Pakistan Medical and Dental Council (PMDC), the regulatory body of medical practitioners has formulated a code of ethics for all doctors, although no concrete steps have been taken to ensure their application [10]. However, most other work on this subject focuses on research ethics and is currently limited to individual institutions or some non-governmental organizations [9]. At the same time, cultural values in Pakistan offer a challenge to the practice of medical ethics in Pakistan. This is because crucial decision making is often done by family members or is left entirely up to the physician, and there seems to be a general acceptance of this shifting of focus from the individual to other people. [11-14]. Public (patient) awareness of their rights to informed consent and privacy is often low [9,14]. Previous qualitative research has shown that a significant number of physicians do not think it is necessary to obtain a proper consent after providing the patients with thorough information [12,14,15]. Furthermore, general observation points to wide differences between the quality of medical care offered at private and public hospitals. In view of these observations, this study was conducted to explore the degree to which the ethical practices of informed consent, privacy and confidentiality are observed in medical outpatient departments of public and private hospitals in Lahore, Pakistan. We follow it up with an assessment of patients' perceptions of these practices in comparison to the assessment performed by our data collectors.

## Materials and methods

A cross-sectional study was conducted at general medical out-patient departments (OPDs) of two tertiary care hospitals of Lahore during the period March-June 2005. One hospital was from the public sector while the other was from the private sector. The sample was selected using multistage random sampling. In the first stage, 6 major public and 4 major private tertiary care hospitals of Lahore were listed separately. (Hospitals were defined as major if they had at least one professor level internist among their list of consultants and if their monthly OPD turnover was > 1000 patients). One hospital was then selected from each list using simple random sampling. Jinnah Hospital was selected from the public sector, while Shalimar Hospital was the private sector hospital included in our study.

In the next stage, the outpatient registration record was used to enroll patients for the study. Records showed that a total of 2800 patients attended the medical OPDs in the two hospitals every month. Therefore, assuming the patient perception of good ethical practices to be 55% [16] and using 0.05 significance level, a sample of 93 patients was required from each hospital. Using systematic random sampling, every tenth patient attending the general medical OPD was selected. In case a patient refused consent, the next patient was approached for the study while sticking to the same interval to select the rest of the patients.

Each patient enrolled in the study was issued a tag for identification. Relevant demographic information was obtained from each patient by a trained data collector (of the same gender as the patient). The same data collector then accompanied the patient during his/her flow through the OPD. These trained data collectors were medical doctors undergoing training in an MPH program. Each doctor-patient interaction was observed and evaluated to fill a peer-reviewed 'client flow analysis form' created in the light of existing literature on the subject [12,17-19]. Each interaction was graded for adherence to the principles of informed consent, privacy and confidentiality.

The assessment of these practices was undertaken in a subjective manner by the data collectors who observed each doctor-patient interaction. It was noted whether doctors took informed consent from their patients before beginning history-taking, beginning physical examination, exposing any part of the body for examination, or discussing treatment options at the end. Confidentiality (informational privacy) was assessed by noting whether there were other people who could potentially overhear the doctor-patient discussion or be told information regarding the patient without prior consent. Taking the patient to another room for examination, or at least taking them behind a screen was categorized as being adequate privacy for the patient.

At the end of the OPD visit, subjects were asked questions on whether they were satisfied with the way these principles were followed by the doctors interacting with them. The three (yes-or-no) questions asked from the patient were whether he/she was satisfied with the doctor's practice of informed consent, privacy and confidentiality. A yes to all three questions was taken to mean that in the patient's perception, the ethical principles had been well observed. However, if the patient thought that at least one of the principles had been followed to his satisfaction, (but not all three) the perception was taken to mean that the ethics had been 'somewhat observed'.

Prior consent had been obtained from all doctors so as to be allowed to observe and evaluate any doctor-patient interaction during the study period. However, in order to minimize bias, at no point were the doctors informed of the individual patient selection. Hence they remained unaware of which patient interaction was being graded for ethical practices. This had been made clear to them while obtaining consent for their participation. In Pakistan, the nursing departments are often understaffed so that the role of nurses in the outpatient departments is limited and it is almost always the doctors who obtain informed consent from the patients regarding their examination/treatment. Therefore, nurses were not included in the study.

Ethical approval for the study was obtained from the review committee of the Center for Health Research, Lahore. The study was conducted in compliance with the 'Ethical Principles for Medical Research involving Human Subjects' of Helsinki Declaration. [20]. Patient names were not recorded to assure confidentiality. Verbal consent was obtained from all subjects and documented in the presence of a witness.

Data was entered and analyzed using Statistical Package for Social Sciences (SPSS) version 12.0. Results were computed separately for each of the two hospitals included in the study. Two sample t-test was employed to detect any significant difference between the public and private hospitals in the practices of informed consent, privacy and confidentiality. Chi square test was employed to detect an association between the patients' perceptions and the observations of our data collectors.

## Results

We enrolled and followed 93 patients in each of the two hospitals. Overall, there were 138 females and 48 males (M:F = 1: 2.8). The mean age of the patients in the public hospital was 34.9 (SD: 15.2, range: 13–79) while that in the private hospital was 37.6 (SD: 15.2, range: 12–79). Other demographic details are shown in Table 1.

**Table 1:** Demographic and Socio Economic Data

	**Public Hospital**	**Private Hospital**
	**Frequency (%)**	**Frequency (%)**

**Gender**		

Female	73 (73.5)	70 (75.3)
Male	20 (21.5)	23 (24.7)

**Age**		

15–25	32 (34.0)	26 (28.3)
25–45	38 (41.1)	41 (44.5)
> 45	23 (24.9)	26 (27.2)

**Profession/Occupation**		

House wives	54 (58.1)	56 (60.2)
Labourer	9 (9.7)	8 (8.6)
Skill Worker	6 (6.5)	8 (8.6)
Student	11 (11.8)	15 (16.1)
Others	13 (14.0)	6 (6.5)

Monthly income/capita in Pak Rs. ($ 1 = Rs 67)*		

< 500	28 (30.1)	15 (16.2)
500–1000	40 (23.2)	58 (62.3)
1000–1500	13 (14.3)	10 (10.8)
> 1500	12 (12.4)	10 (10.7)

Education*		

Illiterate	52 (55.9)	28 (30.1)
< 10 (below matric)	30 (32.3)	46 (49.5)
≥ 10 (matric & above)	11 (11.8)	19 (20.4)

*The differences in the income and educational profile of the patients from the two hospitals reach statistical significance. (p: 0.05 and p < 0.0001 respectively)

Results of adherence to the practice of informed consent, privacy and confidentiality in each hospital are shown in Table 2. Observance of ethical practices was inadequate or improper in most instances. The practice of informed consent in the private hospital was much better compared to the public hospital (p: < 0.0001). No informed consent was taken at all in 90.3% cases in the public hospital compared to 53.3% of the patients in the private hospital. Similarly, confidentiality was adequately practised more often in the private hospital than in the public hospital (p: < 0.0001). On the other hand, the differences in the provision of privacy were not statistically significant.

**Table 2:** Practice of Informed consent, confidentiality and privacy as graded by data collector

	**Public Hospital**	**Private Hospital**	**Two-sample t-test**	
**Informed consent**	**Frequency (%)**	**Frequency (%)**	**T statistic (df:184)**	**p value**

Properly taken	3 (3.2)	2 (2.1)	0.467	0.64
Improperly taken	6 (6.5)	42 (45.7)	6.08	< 0.0001*
Not taken at all	84 (90.3)	49 (53.3)	5.60	< 0.0001*

Total	93 (100)	93 (100)		

**Confidentiality**	**Frequency (%)**	**Frequency (%)**	**T statistic (df:184)**	**p value**

Adequate	10 (10.8)	33 (35.5)	3.993	< 0.0001*
Inadequate	55 (59.1)	41 (44.1)	2.04	0.0421*
Not at all	28 (30.1)	19 (20.4)	1.523	0.1296

Total	93 (100)	93 (100)		

**Privacy**	**Frequency (%)**	**Frequency (%)**	**T statistic (df:107)**	**p value**

Adequate	3 (10.7)	21 (25.9)	1.674	0.097
Inadequate	20 (71.4)	51 (63)	0.80	0.423
Not at all	5 (17.9)	9 (11.1)	0.92	0.356

Total	28^# ^(100)	81^# ^(100)		

*results reach statistical significance

^#^provision of privacy was graded only during physical examinations hence N < 93

Table 3 shows the overall patient perception of the way doctors followed these principles in the OPD of each hospital. Compared to the public hospital, more patients in the private hospital believed that the ethical principles had been well observed by the doctors interacting with them (p: < 0.0003).

**Table 3:** Overall patient perception of observance of ethical principles by doctors

	**Public Hospital**	**Private Hospital**	**Two sample t-test**	
**Patient perception**	**Frequency (%)**	**Frequency (%)**	**T statistic (df:184)**	**p value**

Well observed	1 (1.1)	15 (16.1)	3.64	< 0.0003*
Somewhat observed	66 (71.0)	57 (61.3)	1.39	0.163
Not observed	26 (28.0)	21 (22.6)	0.84	0.398

Total	93 (100)	93 (100)		

*results reach statistical significance

Tables 4 and 5 compare the patients' perception, with the actual adherence/non-adherence to these principles as observed by our trained data collector. The results show that there is an association (p < 0.05) between the perception of the patients and conclusion of the data collector in case of the private hospital (i.e. there is greater concordance). On the other hand, the opinion of the patients and data collectors is not significantly associated (less concordance) in the public hospital (p > 0.05). In the public hospital, the patient perception and the data collector's observation were in agreement in 59.1% of the cases. However, in the private hospital, the patient perception and data collector's observation were in agreement in 76.3% of the cases. In other words, patient perception and data collector's observation were in greater concordance in the private hospital. This difference was statistically significant upon applying a test of two proportions (p: 0.012).

**Table 4:** comparison of the patients' perception, with the actual adherence/non-adherence to ethical principles in the *public hospital *as observed by our trained data collector*

**Actual adherence to ethical principles as concluded by data collector**	**Overall patient perception of observance of ethical principles by doctors**		
	
	Observed	Not Observed	Total
Observed	47	18	65
Not observed	20	8	28

Total	67	26	93

*p value: 0.93

**Table 5:** comparison of the patients' perception, with the actual adherence/non-adherence to ethical principles in the *private hospital *as observed by our trained data collector*

**Actual adherence to ethical principles as concluded by data collector**	**Overall patient perception of observance of ethical principles by doctors**		
	
	Observed	Not Observed	Total
Observed	63	13	76
Not observed	11	8	19

Total	74	21	93

*p value: 0.029

## Discussion

The present study was designed with a purpose to assess the actual practice of informed consent, privacy and confidentiality by the doctors through direct observation of the entire process of patient care provided in outpatient departments (OPDs) of public and private hospitals, and correlate these ethical practices with patient perception of doctors' ethical practices. Our results show that the doctors took proper informed consent from very few patients coming to these hospitals. One of reasons behind such practice is that the cultural trends in Pakistan still tend to accept the paternalistic model of medical care. This is in line with the Asian culture as a whole, where the decision-making is often left purely to the doctors or other family members. Studies from Kashmir and Japan reflect similar practices wherein patients are willing to accept what doctors choose for them, while doctors are satisfied with their role of a decision-maker. [21-23]. For example in a study by Yousaf RM et al [21], 65% physicians in Kashmir and 35% physicians in Malaysia said they would listen to the family's request to withhold information from the patient. A study from Hong Kong also shows the patients and physicians to be more willing to accept the role of families in crucial decisions regarding medical care [24]. Even in countries like Lithuania [25] and South Africa [26], the practices of doctors often do not meet the moral and legal requirements for medical ethics, although the observance of ethics is better than what our study has found in Pakistan.

While the situation in US was not much different till the 1960s [27], the current medical practice in US lays significant focus on the concepts of informed consent and shared decision-making. This differs substantially from the trends in Asia [28] and experts have gone to the extent of calling it a 'cultural artifact' in that reliance on this concept is not universal [29]. Even in US, there is often a clash between these ethical standards and the moral intuitions of many physicians [30,31].

Improper consent of some form was taken from a large number of patients at the private hospital but just a few from public hospital. No informed consent was taken from an alarming proportion of patients (90%) at the public hospital. Even in the private hospital more than half the patients were denied their right to informed consent. On the whole, the practice of informed consent was better at the private hospital but still far from the ideal. Several reasons may account for the differences. Firstly, doctors at private hospitals are better paid than their colleagues in the public sector, something that may translate into better performance at work and greater care for the patients. Secondly, doctors in the private sector are often employed on contracts that need regular renewal. Doctors' work is regularly monitored and assessed, and this renewal is often linked to patient satisfaction with care. Hence doctors in the private sector are more likely to respect the patients' fundamental rights related to their medical management. On the other hand, jobs in the public sector are secure and more or less permanent in nature. At the same time, there is little or no accountability of the doctors since there is usually no effort to elicit patients' opinion about the care provided to them. The results of our study are in line with those from a study conducted in a public sector hospital in Karachi that concluded that the current practice of informed consent was below the internationally acceptable standards [32]. Even though that study commented only on preoperative informed consent, it is pertinent to note that the trend of both our studies is similar. Another study from a private hospital in Karachi also reported that the number of patients complaining of lack of privacy was greater than in the west [33].

Similarly, the principle of confidentiality (informational privacy) was also inadequately practised in our study. This is not surprising since even a study in a country like Canada, has shown that quite a few of the family physicians do not fully understand their obligations towards patient confidentiality [34]. Furhtermore, the practice of confidentialty was more inadequate/unsatisfactory in the public sector hospital than the private one. While the reasons cited above may also contribute to this difference as well, there are others factors that must also be explored. Significant patient burden at general OPDs of public hospitals often makes it impossible for the doctors to follow the full protocol of informed consent and confidentiality. Usually the OPDs are in the form of big rooms in which on one side the patients are waiting (a part of their total waiting time in and outside the OPD room) while on the other, there are some examination tables (with or without a screen). In the center of the room, many doctors are interviewing and examining multiple patients and/or writing medical prescriptions. 2 to 4 patients are dealt with simultaneously. Seldom if ever are the attendants requested to leave the room while the patient is being interviewed or examined. Hence the patient and his/her problems are discussed in front of all present in the room. Such practice may prevent the patients in revealing their complete history and list of symptoms [34].

Provision of privacy during physical examinations was also inadequate in both hospitals. However, privacy-related practices were still somewhat better than the practices of informed consent and informational privacy. The private hospital again showed better ethical practices than the public hospital although in this case the difference was not statistically significant. This may be because in both settings, doctors have no choice but to carry out these examinations behind a screen, especially examinations requiring significant exposure. A study conducted at a private hospital in Karachi also shows that patients felt some lack of privacy on a significant number of occasions (47%) [33]. Our figure in both hospitals is even higher than this. However, socio-demographic differences in the patient population, difference in the method of data collection and the fact that the study in Karachi was carried out on inpatients, precludes any concrete comparison with our results. Imam et al [33] have reported the patient 'opinions' regarding privacy while in our study trained data collectors graded the provision of privacy in comparison to professional standards. As stated earlier, patients can under- or overestimate their ethical rights and hence their opinion may not necessarily be in line with the ideal standards [8,9]. This factor may also contribute to the different figure generated by our study. In comparison to an international study as well, our results show a much greater inadequacy in the provision of privacy to the patients [35].

Our study shows that compared to the public hospital, more patients in the private hospital believed that ethical practices were well observed by doctors interacting with them. This is fairly in line with the assessment of our data collectors where principles of informed consent, informational privacy and physical privacy were more often applied in the private hospitals as discussed earlier. We compared whether the patients' perception of these ethical practices matched correctly with the assessment of our data collectors. In 38/93 instances in the public hospital and 24/93 in the private hospital, patients' perception differed with the assessment of our trained data collector. This is a significant number, and again shows that many patients are unaware of, or misunderstand their ethical rights [8,9]. Once again, the discordance is higher in the public hospital and this may be directly related to the lower socioeconomic status of these patients compared to those in the private hospital.

It is noteworthy, that there are also some other reasons for inadequate ethical practices in Pakistan. For example, although innovative ethical curricula have been shown to improve the confidence and practice of doctors with regards to medical ethics [36], PMDC does not include education in bioethics as a major component of the medical curriculum [14]. It follows, that very few medical colleges in Pakistan impart formal training in bioethics. Such education is also largely omitted from postgraduate training programs. Lack of applied ethical training is also perceived in other countries like Germany [37] and even US [36], which has always championed the cause of bioethics. This lack of Pakistani education in ethics means that trainees can only learn from the practices of their consultants, most of whom belong to the era when a paternalistic approach towards the patients was in vogue. This leads to a vicious cycle where every subsequent generation of doctors believes in paternalism. Even doctors who favor practices like informed consent, often abandon these practices since they believe that most of their patients are uneducated and would not be able to decide what is best for them. It is true though, that often the patients do not want to take any decision and want the doctor to decide each and every thing for them. Furthermore, the lack of accountability and legal recourse means that doctors who do not respect patient ethics are never taken to task in this country.

However, regardless of the excuses provided for the lack of medical ethics, it should be kept in mind that the principles of informed consent, confidentiality and physical privacy must always be applied in medical practice [1,4,6,38].

## Conclusion

Adherence to principles of ethics in medical practice is inadequate in Pakistan. Formal training in bioethics should be incorporated in undergraduate and postgraduate medical training so that the healthcare providers understand the concept, process and application of medical ethics. Local languages should be utilized in written and verbal consent. Forms for written consent should be easy to understand for even the less educated patients. Every patient should be interviewed and examined in a separate room to ensure informational and physical privacy and the number of medical staff should compliment the patient load at any hospital. Sincere attempts need to be made at legalizing the value and processes of medical ethics and public health programs should aim at making the patients aware of their legal rights to informed consent, confidentiality and privacy.

## Competing interests

The authors declare that they have no competing interests.

## Authors' contributions

AH did the overall supervision, participated in the conception of the idea, preparation of the questionnaire and writing of the manuscript. NF was involved in designing the study, and performing data analysis. SN helped in the preparation of the questionnaire and writing the manuscript. SH helped in writing the protocol, data collection, its entry and analysis. AR participated in the conception of the idea, protocol writing, data collection and analysis. HI participated in the conception of the idea, preparation of questionnaire, data collection and entry. SZI was involved in the overall supervision, data analysis and manuscript writing.

## Pre-publication history

The pre-publication history for this paper can be accessed here:


